# Supplementary material for: Mechanisms of sensorimotor adaptation in a hierarchical state feedback control model of speech
Source: PLoS Comput Biol. 2023 Jul 28;19(7):e1011244. doi: 10.1371/journal.pcbi.1011244 (PMC10434967; doi:10.1371/journal.pcbi.1011244)
Supplement: S1 Appendix — (DOCX) [file pcbi.1011244.s001.docx]

**Mechanisms of sensorimotor adaptation in a hierarchical state feedback control model of speech**

Kwang S. Kim, Jessica L. Gaines, Benjamin Parrell, Vikram Ramanarayanan, Srikantan S. Nagarajan, John F. Houde

**S1 Appendix.**

We conducted further analyses to examine why Design A shows little to no adaptation. One possibility is that the articulatory state estimate updates in some parameters (e.g., the Jaw Height) may be non-optimal. As a result, some articulatory estimates can be misestimated in Design A, likely contributing to slight auditory prediction changes towards “maladaptive” direction (see Fig 3 in the main manuscript). This phenomenon seems to be caused by the articulatory state prediction being implemented as multiple LWPR models each functioning as an integrator for a single articulatory dimension (e.g., Jaw Height). Thus, it is possible that the model for Jaw height may be (incorrectly) influenced more by somatosensory feedback than auditory feedback during adaptation.

Another possibility is that because the articulatory state prediction LWPR functions as an integrator for the efference copy (articulatory acceleration), changes in the learned model may be limited to very local contexts. This would be reflected in the model by a large number of receptive fields, each covering only a small area of the input space. However, our analyses showed a the LWPR models contain a very small number of receptive fields, suggesting that the learning is rather broad rather than overly-local. We conducted a series of additional simulations to determine whether somatosensory signals may have limited the amount of adaptation in Design A. Interestingly, we found that by eliminating or reducing the somatosensory feedback in the state correction (i.e., the articulatory state estimate is corrected based on only the auditory feedback), it is indeed possible to induce a behavior that, at first glance, somewhat resembles learning. Nevertheless, the model behavior is unlike adaptation reported by previous studies in that this design’s adaptive changes are found almost exclusively in the later vowel portions, with minimal changes seen at vowel onset (see Fig A in S1 Appendix).


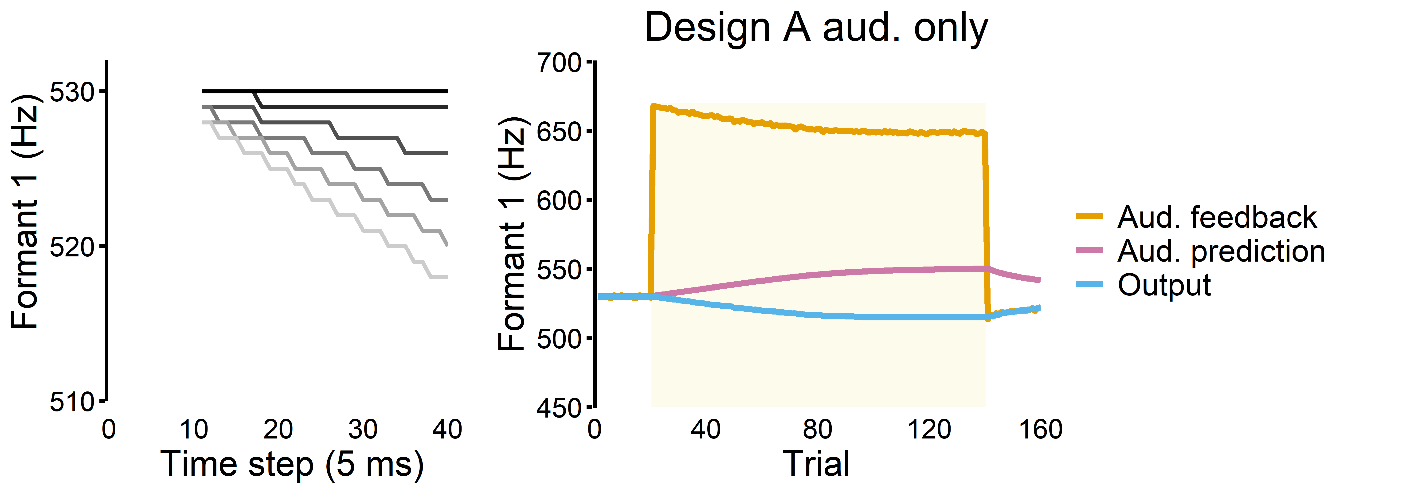


**Fig A.** Design A with no somatosensory feedback in the state correction. **Left:** Formant trajectories across time steps for trials 20, 22, 24, 26, 28, and 30 (shown as darker shade to lighter shade). Perturbation was set to begin on trial 21. Compared to the baseline trial (trial 20), other trials show accelerating behaviors. **Right:** If the formant frequencies were extracted from the early vowel portion (time step 11-13), the changes in the first formant frequency are minimal across the trials.

The ever-accelerating behavior (i.e., velocity increases without reaching a stable position as in Design C) seems to be a sign not of adaptive learning, but rather of unstable control. This instability agrees with simulation results from our initial paper on FACTS, where we found that minimizing or removing somatosensory feedback results in an unstable control in FACTS [1]. Therefore, although it may be possible for Design A to induce adaptation if the relationship between the motor commands and the articulatory state were implemented differently, the articulatory state estimate diverging from the actual state may also introduce some instability. Importantly, this behavior was also observed when the model was implemented with an adaptive UKF (AUKF, see related implementation in Design C in the main text). In addition, even though implementing an AUKF in Design C resulted in a realistic adaptation simulation, the same implementation did not yield adaptation in Design A (see Fig B in S1 Appendix).


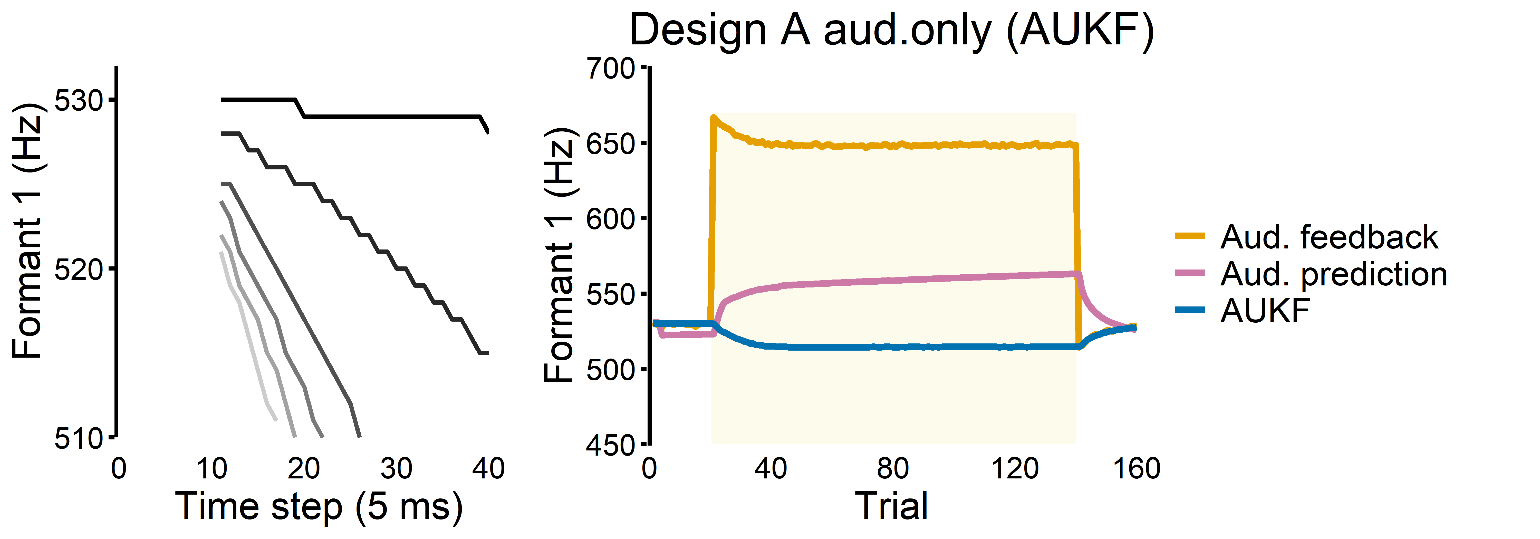


**Fig B.** Design A (with no somatosensory feedback state correction) implemented with AUKF. **Left:** Formant trajectories across time steps for trials 20, 22, 24, 26, 28, and 30 (shown as darker shade to lighter shade). AUKF did not alter the accelerating behaviors. **Right:** The formant frequencies extracted from the early vowel portion (time step 11-13) showed minimal changes across the trials, suggesting that AUKF did not increase adaptation.

References

1. Parrell, B., Ramanarayanan, V., Nagarajan, S., & Houde, J. (2019). The FACTS model of speech motor control: Fusing state estimation and task-based control. PLoS computational biology, 15(9), e1007321. https://doi.org/10.1371/journal.pcbi.1007321
