# Supplementary material for: Mechanisms of sensorimotor adaptation in a hierarchical state feedback control model of speech
Source: PLoS Comput Biol. 2023 Jul 28;19(7):e1011244. doi: 10.1371/journal.pcbi.1011244 (PMC10434967; doi:10.1371/journal.pcbi.1011244)
Supplement: S2 Appendix — (DOCX) [file pcbi.1011244.s002.docx]

**Mechanisms of sensorimotor adaptation in a hierarchical state feedback control model of speech**

Kwang S. Kim, Jessica L. Gaines, Benjamin Parrell, Vikram Ramanarayanan, Srikantan S. Nagarajan, John F. Houde

**S2 Appendix.**

During the FACTS development, the initial simulations indicated that the resolution of the default Maeda model could be low (e.g., 8.5 Hz in F1). That is, even when the Maeda input parameters were changed by a small amount (e.g., incremented by 0.02 Maeda Unit), the resulting Maeda outputs of F1 and F2 either did not change for multiple steps or jumped by 8.5 Hz (see “Default Maeda” in the top left of Fig A in S2 Appendix). The low resolution of the formant frequencies can also be observed in the big data sets generated by a random walk algorithm in which the Maeda inputs were randomly increased or decreased by a small amount in each step [1]. In theory, the Maeda outputs from such big data sets should completely cover the vowel space. However, even with such a large number of data points, there were still clearly noticeable gaps that appeared in various areas in the F1 range, resulting from the lack of data in those areas (see white striations the left panel in Fig B in S2 Appendix). Although the similar gaps in F2 cannot be observed from the figure because of the larger scale of F2, the F2 resolution was not better than that of F1, as shown in the top right panel in Fig A in S2 Appendix (pink).


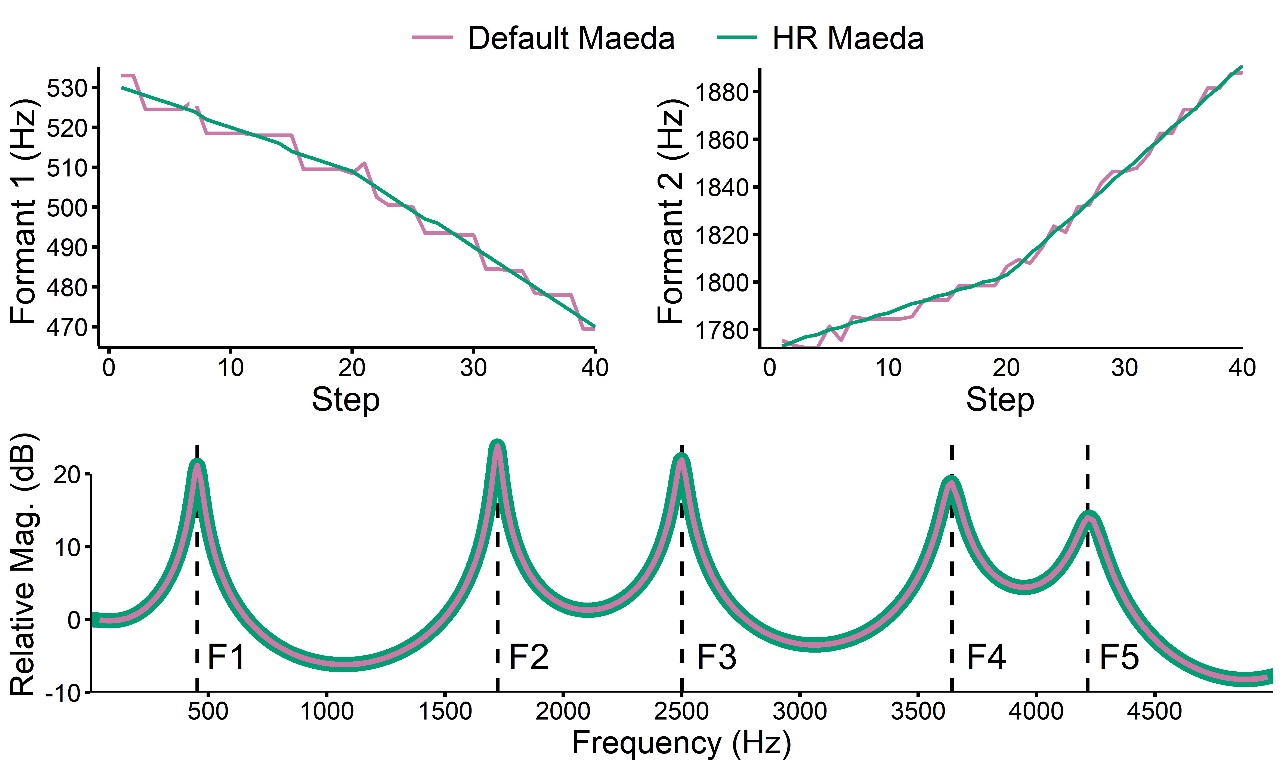


**Fig A.** **Maeda resolution in F1 and F2. Top:** The Maeda input parameter for the Jaw height was incremented by 0.02 (M) in the first 20 steps. During the latter 20 steps, the input parameter for the Tongue height was decremented by 0.02. All other input parameters were kept at 0. The HR Maeda showed higher resolution in both F1 and F2 across these steps. **Bottom**: The transfer function of the default and HR Maeda. The HR Maeda did not drastically differ from the original model in all formant frequencies, bandwidths, and magnitudes. Because of the two transfer functions were very similar to each other, the HR Maeda (in green) was plotted with a larger thickness to avoid a complete overlap. Note that the small differences (< 7 Hz) in the formant frequencies that result from resolution changes cannot be seen in this figure because of the large scale (0 to 5,000 Hz).

**
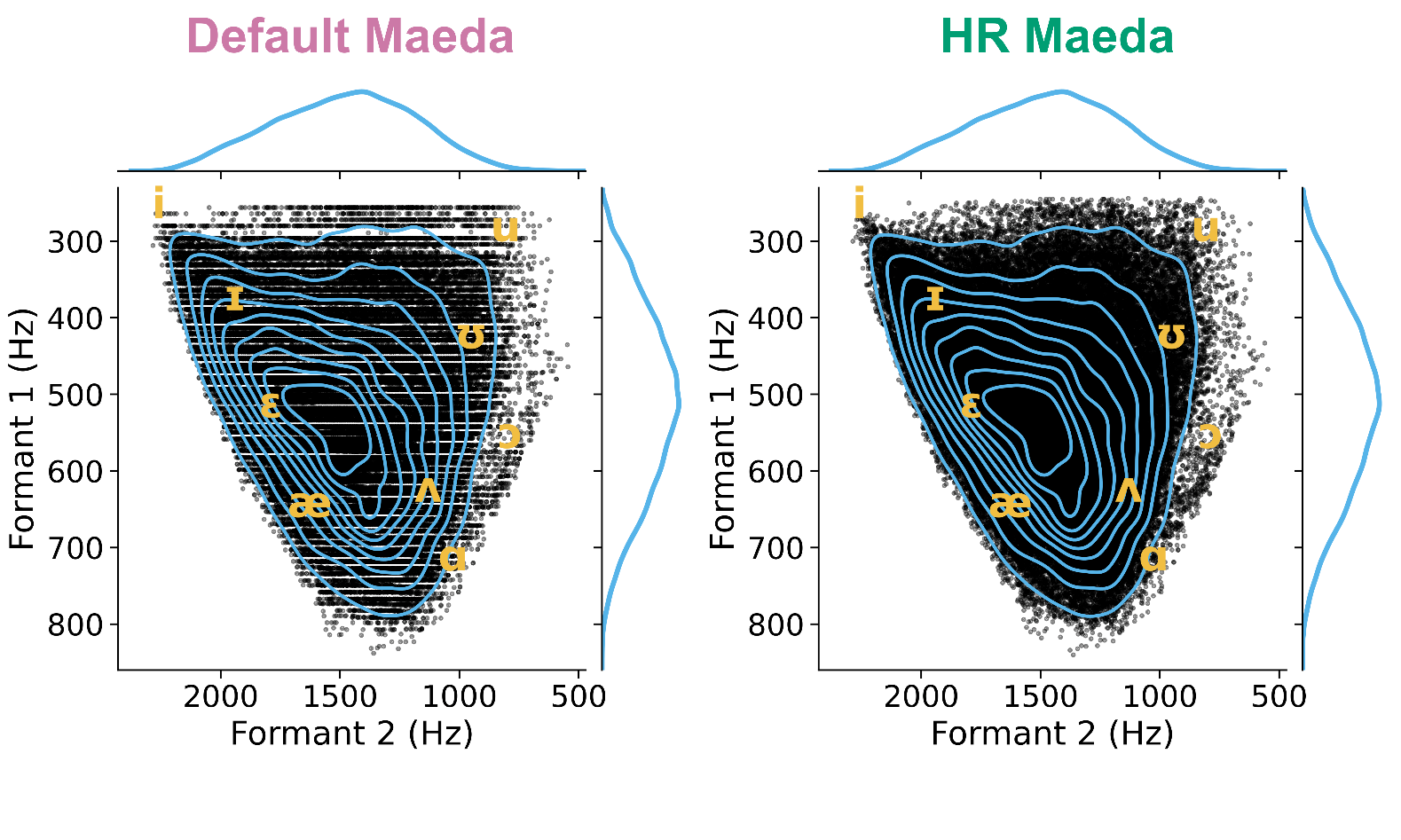
**

**Fig B. Randomly generated Maeda data in vowel space.** A random walk algorithm similar to Gaines et al. [1] was employed to generate a large data set (100,000) of Maeda input parameters. A subset (84,046) of the input parameter data set was chosen given their likelihood to generate valid formant frequencies (i.e., not too close to the maximum or minimum input space). Although both the left and right panels were generated using the same data subset, the HR Maeda shows more complete coverage of the vowel space, without any visible data gaps. The similar improvement with a higher resolution can also be observed in the second formant frequencies (x-axis). Importantly, the overall distribution did not differ between the HR Maeda and the default Maeda, providing evidence that the main difference between the two models was in the resolution-related, relatively small changes in the formant frequencies.

Upon inspecting the Maeda source code, we realized that the low resolution was a byproduct of a formant searching algorithm that was designed to reduce computational costs. The algorithm starts to search for formant peaks initially with a large frequency step (i.e., 40 Hz) and varies frequency step sizes by 8 Hz during the operation. Thus, the frequency step size could be as small as 0.5 Hz or as large as 40 Hz during the formant peak search operation, resulting in some computed formant frequencies with low resolutions. Given that the Maeda model was originally developed several decades ago, such computational cost saving methodology was presumably necessary to operate within the then-available computing power.

In order for FACTS to simulate some experimental data of small changes in formant frequencies such as online compensation (Fig. 6 in the main text), a higher resolution of acoustic output was desired. Therefore, we modified the Maeda source code, specifically to generate formant frequencies with a higher resolution. In the source code method that searches formant peaks (“calplot_tf_FBA”), we fixed the frequency step size to 1 Hz and removed the varying step size algorithm. As a result, the modified Maeda (High resolution Maeda, or “HR Maeda”) model searched for formant peaks at every Hz, generating a higher resolution of 1 Hz. The output data demonstrates much finer-grade F1 and F2 changes in response to small input changes (Fig A in S2 Appendix, top). The HR Maeda model’s improved performance can also be seen in the right panel in Fig B in S2 Appendix, in which all the white striations (or data gaps) are completely absent.

Despite the performance improvement, the modification did not result in any large systematic output changes from the original Maeda. The only differences in the output were merely a difference of 4-5 Hz as a result of the resolution changes. In fact, the transfer function of the HR Maeda was nearly identical to the default Maeda (see the bottom panel in Fig A in S2 Appendix,), demonstrating that formant frequencies, bandwidths, and magnitudes all remained nearly the same. Because the HR Maeda model behaves very similarly with the original Maeda (other than the improvement in the resolution), it can easily replace the original Maeda model in future modelling work as well as various models that are already established.

Lastly, the HR Maeda did require higher computational costs, taking 68.49 s (SD = 0.86 s) to generate 25,000 Maeda model computations on average (n = 5 tests), much longer than the default model which took 28.09 s (SD = 2.04 s). However, such difference can be considered negligible given that most of our model simulations, including adaptation simulations, require far less than 25,000 computations. In the current study, therefore, the HR Maeda was implemented as the plant module. The HR Maeda source code is also now publicly available on the GitHub repository [2].

References

1. Gaines, J. L., Kim, K. S., Parrell, B., Ramanarayanan, V., Nagarajan, S. S., & Houde, J. F. (2021). Discrete constriction locations describe a comprehensive range of vocal tract shapes in the Maeda model. *JASA express letters*, *1*(12), 124402. <https://doi.org/10.1121/10.0009058>
2. Ghosh S. (2013). VocalTractModels. Available from: https://github.com/satra/VocalTractModels.
