# Supplementary material for: Mechanisms of sensorimotor adaptation in a hierarchical state feedback control model of speech
Source: PLoS Comput Biol. 2023 Jul 28;19(7):e1011244. doi: 10.1371/journal.pcbi.1011244 (PMC10434967; doi:10.1371/journal.pcbi.1011244)
Supplement: S3 Appendix — (DOCX) [file pcbi.1011244.s003.docx]

**Mechanisms of sensorimotor adaptation in a hierarchical state feedback control model of speech**

Kwang S. Kim, Jessica L. Gaines, Benjamin Parrell, Vikram Ramanarayanan, Srikantan S. Nagarajan, John F. Houde

**S3 Appendix.**

The LWPR model can be tuned to be more “forgetful” (i.e., forgetting the previously learned input-output relationships and learning the new ones faster) or less “forgetful” by changing forgetting factors. There are three forgetting factors in LWPR: *init lambda*, *tau lambda*, and *final lambda*. The forgetting factor starts with *init lambda* and “anneals” towards final lambda with an annealing constant tau lambda. The default parameters included in the LWPR manual [1] are: 0.995 (*init lambda*), 0.9999 (*tau lambda*), and 0.99999 (*final lambda*). Here, if the forgetting factor is closer to 1, it means the LWPR model is less “forgetful”. All simulations included in the results section were done with forgetting factors close to these default values: 0.985 (*init lambda*), 0.995 (*tau lambda*), and 0.99995 (*final lambda*).

These parameters can affect the adaptation rate and extent (see Fig A in S3 Appendix, A) “more forgetful” model). However, they display the classic stability versus plasticity dilemma (e.g., [2]). That is, if a LWPR model is tuned to be too “forgetful” (so that it learns more and faster), it loses stability. Indeed, even when a LWPR model was tuned to be slightly more forgetful than the default setting listed above, the model’s instability often caused FACTS simulations to halt (see Fig A in S3 Appendix, A). In addition, the forgetting factors’ effects on adaptation were minimal in Design C implemented with AUKF.


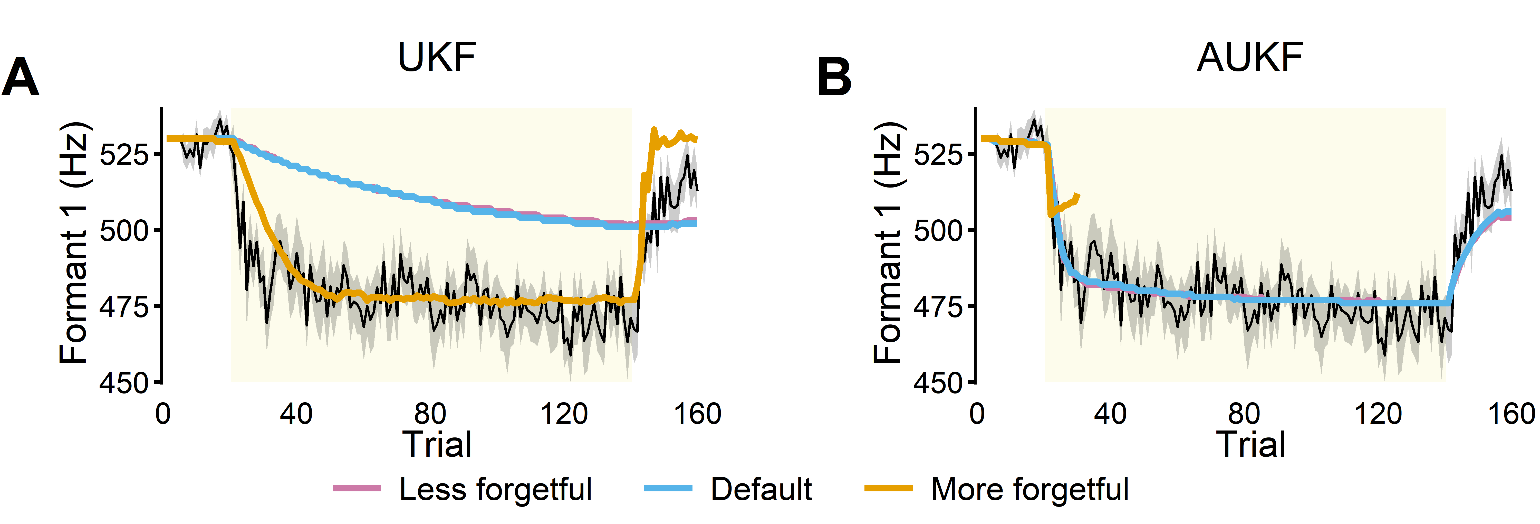


**Fig A.** **The effect of changing forgetting factors in LWPR on FACTS adaptation.** In both A and B the “Less forgetful” simulations shown in pink had 0.9995 (*init lambda*), 0.999999 (*tau lambda*), and 0.9999995 (*final lambda*). For the “more forgetful” simulations shown in orange, forgetting factors were: 0.97 (*init lambda*), 0.98 (*tau lambda*), and 0.985 (*final lambda*). The “Default” simulations shown in blue had the same forgetting factors used in the simulations (0.985 (*init lambda*), 0.995 (*tau lambda*), and 0.99995 (*final lambda*)). **A**: In Design C with UKF, more “forgetful” LWPR helped the FACTS model to achieve larger adaptation extent and rate. Nevertheless, the initial learning was much slower than the empirical data reported by Kim & Max [3]. Moreover, the instability of LWPR caused FACTS to break at trial 143 (see “more forgetful” shown in orange). **B**: In Design C with AUKF, the forgetting factors had minimal effects. Again, “more forgetful” model stopped in the middle of the simulation (around trial 95) due to the instability of LWPR.

References

1. Vijayakumar S. Locally Weighted Projection Regression (LWPR) - a users manual; 2001. Available from: https://homepages.inf.ed.ac.uk/svijayak/software/LWPR/LWPRmanual.pdf
2. Abraham, W. C., & Robins, A. (2005). Memory retention--the synaptic stability versus plasticity dilemma. Trends in neurosciences, 28(2), 73–78. <https://doi.org/10.1016/j.tins.2004.12.003>
3. Kim, K. S., & Max, L. (2021). Speech auditory-motor adaptation to formant-shifted feedback lacks an explicit component: Reduced adaptation in adults who stutter reflects limitations in implicit sensorimotor learning. The European journal of neuroscience, 53(9), 3093–3108. <https://doi.org/10.1111/ejn.15175>
