# Supplementary material for: Mechanisms of sensorimotor adaptation in a hierarchical state feedback control model of speech
Source: PLoS Comput Biol. 2023 Jul 28;19(7):e1011244. doi: 10.1371/journal.pcbi.1011244 (PMC10434967; doi:10.1371/journal.pcbi.1011244)
Supplement: Attachment — Submitted filename: Review_PCOMPBIOL-D-22-01400.pdf [file pcbi.1011244.s004.pdf]

I. Overview

The manuscript presents, in the context of the FACTS model of speech production, a study of adaptation mechanisms in speech production when auditory feedback is artificially altered. Inspired by Kim & Max (2021) auditory perturbation experiment, the authors attempted to simulate adaptation in their model when a 400 cents up-shift was applied to formant F1 during 120 short repetitions (150ms duration) of vowel /ɛ/. More specifically three variants of the FACTS models were evaluated, which differ from each other in the nature of the internal model that is updated during adaptation. In design A, the internal model that estimates the articulatory state from the efference copy of the articulatory motor command was updated; in design B the internal model that predicts the acoustic output from the articulatory state was updated; in design C, the original architecture of the FACTS model was modified: the estimation of the task state (in this model the characteristics of the vocal tract constriction), which was originally only based on the estimation of the articulatory state provided by a Kalman filter using both somatosensory and auditory feedbacks, is now based on a new internal model that predicts the task state from the estimated articulatory state via a Kalman filter based on auditory feedback only. In this design adaptation updates Based on several results of the literature suggesting that there is no, or very little, auditory feedback-based corrections during vowel productions lasting less than 150ms, online feedback-based corrections were disabled in all the three designs during the production of each repetition of the vowel. Nevertheless, in each design the output of the updated internal model was stored in a memory if the difference between the predicted acoustic feedback resulting from this update and the actual altered acoustic feedback was larger than a given threshold, this stored information was used to update the model in the subsequent repetition of the vowel.
Adaptation is evaluated on the basis of whether or not the actual altered acoustic feedback is modified along the repetitions of the vowel so as to reach the formant values usually associated with vowel /ɛ/. It is found that Designs A and B do not generate any adaptation. Only design C generates adaptation and the trends of the simulated adaptation are similar to those observed in Kim & Max (2021) study with human subjects. However, the authors found in their simulations a significantly lower magnitude of adaptation than in the experimental data, which they attributed to the limitations of the Kalman filter, which tends to attribute large discrepancies between predicted and actual feedback primarily to noise added to the actual feedback rather than to errors in the feedback predictions. In order to overcome these limitations they implemented an adaptive Kalman filter (Adaptive Unscented Kalman Filter, AUKF), with which they obtained adaptation magnitude very close to the one observed in experimental data.
With this final design, the authors (1) reproduced after-effects and unlearning after the removal of the auditory feedback, (2) investigated the role of auditory prediction in adaptation by comparing a model in which auditory prediction follows the changes in task state estimation and a model in which auditory prediction remains unchanged, (3) discussed possible origins of incomplete adaptation to auditory feedback alteration usually observed in experimental studies, and considered possible origins of inter-trial variability in relations with noise on the specification of the task targets. They also showed that the new model, i.e. Design C with AUKF, reproduces the experimentally observed online compensation of unpredictable formant perturbation, which was an important result obtained with the original variant of FACTS.
The main conclusion of the authors is that, contrary to a hypothesis that is often proposed in the literature, updates in internal models are not crucial for adaptation in speech motor control. They rather suggest that updates in control policy, i.e. in the case of their FACTS model in the task state feedback control law that assesses the discrepancy between the task state estimate and the task target, is crucial. This update can involve update in the internal model that estimates the task state from the efference copy of the motor commands.

The manuscript tackles a number of important issues in speech control motor. The paper is well-structured, and in most of the sections precisely and clearly written. The addressed topics are interesting primarily for the research community working on speech production and speech perception, but also more generally for all the scientists interested in adaptation mechanisms

in human motor control. It also represents an important amount of work. The willingness to share the results of this research work by making programs written in Python available to the community also deserves to be highlighted. However, in our opinion, at this stage, additional information and clarification are needed for a better understanding of the proposed modeling work and, then, to better assess the relevance of the main conclusions. Hence, at this stage, major modifications are required. They do not necessarily require a significant amount of work, but they are in our opinion required for the publication to be considered.

II. Major points

The first point to be clarified is the behavior of Design A.
The authors wrote that in all three designs "*the feedback pathways for online compensation were disabled and direct pathways from articulatory and task state prediction systems were used instead*" (lines 133-134). Thus adaptation is modelled from trial to trial by saving the updated predictive/transformation models of trial n and by using them on trial (n+1) (lines 143-146). However, this strategy does not seem to be systematic, since "*auditory feedback or its related information (i.e., the final state estimate updated by state correction signals in Design A and C) were stored in an "error memory,* [only] *if the auditory prediction errors were larger than a given threshold.*" (lines 1043-1045). The authors also added: "*We also tested the model simulations with online auditory and somatosensory feedback, but the results were comparable with the simulations presented in the study (i.e., no sensory feedback for guiding movements online).*"
In this context, the results obtained with design A, as presented in the left panels of Figure 3, require further explanations. Indeed the bottom left panel shows very significant changes in the predicted articulatory state during trials 22, 24, 26, 28, and 30, whereas the difference between the auditory feedback and the auditory prediction remains quasi unchanged across these trials and in any case exceeds the auditory prediction error threshold considered in this study (5Hz, 30Hz, or 50Hz, according to Figure7). According to the above description, if the changes in the predicted articulatory state are associated with a significant update of the internal model that generates the estimation of the articulatory state, the integration of this update from a trial to the next should induce significant changes in the predicted articulatory state (similar to those given in Figure 3) for the unchanged motor commands and this should induce changes in the estimated task state (via the orange box "Task state estimator" in Figure 1) and changes in the output of the "Articulatory state feedback control law". In turn these changes should induce changes in motor commands and then changes in the auditory output of the model. How to explain then that no adaptation, i.e. no change in motor commands and then no change in auditory output is observed? This question is all the more crucial, since the author add that even if the online compensation mechanism is active no adaptation is observed. Since the authors have shown in their former paper (ref 38 in the manuscript) that their model is able to account for online compensation to unpredicted formant perturbation, the problem does not seem to lie in the correction loops. Based on the bottom left panel of Figure 3, it can be considered that the internal model update is taken into account from a trial to the next, since the tongue elevation is different across trials; from trial 10 where this update starts. In addition to the fact that this update from trial to trial does not seem to have any effect on the task state estimation, it is observed that the slope of change in the articulatory state estimation increases from trial to trial, which let the authors speak about "instability". Hence, all the results presented in the left panels of Figure 3 for Design A, require better analysis and explanations. A possible explanation for this somewhat strange behavior could lie in the characteristics of the internal model that estimates the articulatory state and in its update. The calculation of the predicted articulatory state is given in Equation 8 (lines 942-943). It seems to correspond to the integration of a first order ordinary differential equation with constant time steps, which is functionally accounted for via LWPR learning. During a trial with altered auditory feedback, the gradual shift of the articulatory state induced by the Kalman filter is probably associated with a limited local learning of the (first order) regression in a part of the space that is considered to be a so far unexplored region. Could it be that such a local learning is too limited to properly

account for the required changes in articulatory state estimation in presence of the applied auditory feedback perturbation? This nevertheless would not explain why the actual tongue state would tend to (very slowly) drift towards the maladaptive direction (Figure 3, left column, top panel, blue line).
There are additional questions related to the behavior of Design A as described in Figure 3. Why do the changes in predicted articulatory state not induce changes in auditory prediction, or rather, minute changes in the maladaptive direction (Figure 3, left column, top panel)? Similarly, changes in articulatory-state should result in a change of the predicted somatosensory feedback and then in a somatosensory prediction error. This somatosensory prediction error should induce a limitation of the articulatory state changes induced by the auditory prediction error. However, according to Figure 3, it does not seem to be the case. How to explain these results. Are both sensory prediction errors taken into account with different weights? Finally why did the authors interpret the articulatory state changes depicted in Figure 3 as "instability" (See caption of Figure 3) whereas they tend to reflect proper Kalman integration of a biased feedback in a system that does not learn?

The second point concerns Design B. In this design, the update concerns only the auditory prediction: the Kalman filter induces changes in the relationship between the articulatory state and the predicted auditory feedback so as to match the predicted feedback to the altered auditory feedback. This update induces no change in the predicted articulatory state, which in turn should, on the next trial, induce no change in the estimated task state and in the output of the articulatory state feedback control law. Therefore, there is no reason for motor commands to change and no adaptation is observed. However, as a result the auditory feedback is not at all what is expected for the intended vowel /ɛ/. It seems strange to suggest that solely this auditory prediction would update, as if auditory prediction error were entirely ascribed to a problem in the auditory feedback pathway! In fact the behavior of Design B could be deduced from a simple analysis of how the Kalman filter works in this design. We therefore wonder whether the presentation of this design is really relevant from the point of view of speech motor control, unless the authors want to show the limitations of implementing a Kalman filter in this context. In particular, these results do not seem to discard the general relevance in adaptation of the update of an internal model that predicts auditory feedback. Indeed, it is quite likely that a similar implementation of the Kalman filter in a model where the target would be in auditory space, and not in the constriction space of the vocal tract, would induce an adaptation. Indeed Tourville and colleagues mentioned successful adaptation modeling based on such an update in the DIVA model (Tourville, J. A., Reilly, K. J., & Guenther, F. H. (2008). Neural mechanisms underlying auditory feedback control of speech. *Neuroimage*, *39*(3), 1429-1443) and Patri and colleagues have also modeled in this way adaptation to altered auditory feedback in the GEPPETO model (Patri, J. F., Diard, J., & Perrier, P. (2019). Modeling sensory preference in speech motor planning: a Bayesian modeling framework. *Frontiers in Psychology*, *10*, 2339), and more generally in any model in which auditory targets contribute to the specification of the task (Patri, J. F., Perrier, P., Schwartz, J. L., & Diard, J. (2018). What drives the perceptual change resulting from speech motor adaptation? Evaluation of hypotheses in a Bayesian modeling framework. *PLoS computational biology*, *14*(1), e1005942).
More generally the whole paragraph that is devoted to the evaluation of the role of auditory prediction (lines 278-293 and Figure 5) could benefit from being more nuanced and limiting the interpretations to the strict framework of the model and to the scope of evaluation used in this manuscript. For example the authors observed that adaptation was faster when in Design C they "*artificially fixed the auditory prediction to remain at the baseline value*" (line 284), in other words when the auditory prediction does not respond to the changes in the predicted task state induced by the Kalman filter. This is quite a straightforward consequence of the way the Kalman filter works: ignoring the consequence of adaptation in the auditory prediction, will tend to maintain large auditory prediction errors and then strong feedback based correction. However, does it not generate a risk of instability in adaptation, with a possibly infinitely long adaptation due to the risk that the auditory-prediction error could remain large? More generally

the proposed analysis of the role of auditory prediction in adaptation is only valid for the task specification used in the model. As mentioned above with the results obtained with the DIVA and the GEPPETO models, it can be expected that any other model in which the task would be specified in the auditory domain would lead to different conclusions. It would be nice to consider more explicitly this alternative perspective.

The third point concerns Design C. The results obtained with this design are great and this is clearly to the credit of the authors. However their proposed interpretation requires further justification. The authors wrote: "[…] *although the mathematical compositions of the task SFC law does not change as a result of the task transformation updates, the output of the task SFC law change due to the updates in the transformation. Following this line of reasoning, it could be argued that the core change that yielded adaptation was the control policy (i.e., task SFC law) being "updated" per se.*" (lines 478-482). As a matter of fact, this is not fully correct: the control policy does not change; but the predicted task state changes. According to the above mentioned studies in DIVA and GEPPETO adaptation to altered auditory feedback can be properly modeled if the task space is in the auditory space, and if feedback-based correction changes the predicted auditory feedback, which does not affect in any way the control policy. Hence, we rather believe that the results obtained with Design C, as compared to Design A (Design B being not really relevant in our opinion, see above), are due to the fact that the update applies to the internal model that predicts the task state, whether it be constrictions in the vocal tract as in FACTS or regions in formants space as in DIVA or GEPPETO. Furthermore, Design A could give similar results as Design C, since task state depends on articulatory state, if the relationship between the motor commands and the articulatory state were not as simplified as they are in FACTS, thus precluding significant plasticity at this level. If the authors disagree it would be good if they could provide more convincing support for their interpretation.

III. Minor points

Page 3, lines 48-49.
The authors wrote: "*However, errors can drive adaptation even in the absence of corrective movements in both speech [7] and non-speech [32, 33] domains*."
This sentence could be clarified. In [7] and [32] the authors considered online corrections. Movement corrections did occur from trial to trial. In [33] movements were planned, and the results do not discard the possibility that the efference copy of motor commands was corrected to modify sensory predictions.

Page 5, lines 167-170.
The authors wrote: "*The articulatory state data across time steps showed that the articulatory state estimates started to diverge from the true state as the forward articulatory prediction model was updated (Fig 3, bottom left), likely driven by the up-shift F1 perturbation causing the estimator to under-estimate the true tongue height*."
First it would be helpful in all the bottom panels of this figure to know the link between the parameter value in the Y-axis and the tongue height. Since the bottom right figure illustrates adaptation to the F1 up-shift, it can be guessed that a decrease of the parameter is associated with an elevation of the tongue, which is the way to counteract F1 increase.
In this context, the general trend of the results represented in the bottom-left figure 3 makes sense: the articulatory state estimated via the Kalman filter changes so as to lower the tongue in order for predicted auditory feedback to get closer to the altered actual auditory feedback. Hence, why do the authors consider that the estimator "*under-estimate the true tongue height*"?

Page 15, lines 537-540.
The authors wrote: "*In other words, adaptation extent in FACTS was limited by changes in auditory prediction and its error threshold. To our knowledge, this Idea has never been examined before and remains to be investigated in future studies*."
Is the concept of auditory error threshold different from the concept auditory acuity in speech perception?

Page 15, lines 561-567
The authors wrote: "*Second, there may be biomechanical reasons why changing the tongue position to counter the F1 shift also changes F2. However, this latter point is not supported by our previous study with the Maeda model that showed a nearly full coverage of the vowel space in the Maeda model output ([78] and also see S1 Appendix). This means that, at least for the target vowel used in the current study (/E/), the adaptive behavior in FACTS should not be constrained by the kinematics of the Maeda model (i.e., articulatory-level changes can certainly be made to alter F1, but not F2).*"
We understand the line of thinking of the authors. However, as a matter of fact, studies of sensitivity functions in the vocal tract show that any change in vocal tract area function induces simultaneous changes in F1, F2 and F3…

Page 16, section "Inter-trial variability »
It would be interesting if the authors could tell more about the conceptual difference between the notion of target noise and the notion of target regions as used in DIVA or GEPPETO.
