# Supplementary material for: Mechanisms of sensorimotor adaptation in a hierarchical state feedback control model of speech
Source: PLoS Comput Biol. 2023 Jul 28;19(7):e1011244. doi: 10.1371/journal.pcbi.1011244 (PMC10434967; doi:10.1371/journal.pcbi.1011244)
Supplement: Attachment — Submitted filename: ReplytoReview_Kim_Mechanisms_PCOMPBIOL.docx [file pcbi.1011244.s005.docx]

**Authors’ responses to the review of PCOMPBIOL-D-22-01400, Mechanisms of sensorimotor adaptation in a hierarchical state feedback control model of speech. Corresponding Author: Kwang S. Kim, Ph.D.**

Note: Below, all of the editor’s and reviewers’ comments are reproduced verbatim in non-indented paragraphs with *blue italicized font*. Our responses are provided with black non-italicized font.

*Dear Kwang and colleagues,*

*You will find a thorough review by two experts in the field of your manuscript. Note that the GitHub link you provided was not functional. We look forward to your detailed responses.*

*Best wishes,*

*Frederic Theunissen*

Thank you for the thorough reviews. We updated the GitHub repository link, which is now functional.

*Reviewer's Responses to Questions*

*Comments to the Authors:*

*Please note here if the review is uploaded as an attachment.*

*Reviewer #1: The manuscript presents, in the context of the FACTS model of speech production, a study of adaptation mechanisms in speech production when auditory feedback is artificially altered. Inspired by Kim & Max (2021) auditory perturbation experiment, the authors attempted to simulate adaptation in their model when a 400 cents up-shift was applied to formant F1 during 120 short repetitions (150ms duration) of vowel /ε/. More specifically three variants of the FACTS models were evaluated, which differ from each other in the nature of the internal model that is updated during adaptation. In design A, the internal model that estimates the articulatory state from the efference copy of the articulatory motor command was updated; in design B the internal model that predicts the acoustic output from the articulatory state was updated; in design C, the original architecture of the FACTS model was modified: the estimation of the task state (in this model the characteristics of the vocal tract constriction), which was originally only based on the estimation of the articulatory state provided by a Kalman filter using both somatosensory and auditory feedbacks, is now based on a new internal model that predicts the task state from the estimated articulatory state via a Kalman filter based on auditory feedback only. In this design adaptation updates Based on several results of the literature suggesting that there is no, or very little, auditory feedback-based corrections during vowel productions lasting less than 150ms, online feedback-based corrections were disabled in all the three designs during the production of each repetition of the vowel. Nevertheless, in each design the output of the updated internal model was stored in a memory if the difference between the predicted acoustic feedback resulting from this update and the actual altered acoustic feedback was larger than a given threshold, this stored information was used to update the model in the subsequent repetition of the vowel.*

*Adaptation is evaluated on the basis of whether or not the actual altered acoustic feedback is modified along the repetitions of the vowel so as to reach the formant values usually associated with vowel /ε/. It is found that Designs A and B do not generate any adaptation. Only design C generates adaptation and the trends of the simulated adaptation are similar to those observed in Kim & Max (2021) study with human subjects. However, the authors found in their simulations a significantly lower magnitude of adaptation than in the experimental data, which they attributed to the limitations of the Kalman filter, which tends to attribute large discrepancies between predicted and actual feedback primarily to noise added to the actual feedback rather than to errors in the feedback predictions. In order to overcome these limitations they implemented an adaptive Kalman filter (Adaptive Unscented Kalman Filter, AUKF), with which they obtained adaptation magnitude very close to the one observed in experimental data. With this final design, the authors (1) reproduced after-effects and unlearning after the removal of the auditory feedback, (2) investigated the role of auditory prediction in adaptation by comparing a model in which auditory prediction follows the changes in task state estimation and a model in which auditory prediction remains unchanged, (3) discussed possible origins of incomplete adaptation to auditory feedback alteration usually observed in experimental studies, and considered possible origins of inter-trial variability in relations with noise on the specification of the task targets. They also showed that the new model, i.e. Design C with AUKF, reproduces the experimentally observed online compensation of unpredictable formant perturbation, which was an important result obtained with the original variant of FACTS.*

*The main conclusion of the authors is that, contrary to a hypothesis that is often proposed in the literature, updates in internal models are not crucial for adaptation in speech motor control. They rather suggest that updates in control policy, i.e. in the case of their FACTS model in the task state feedback control law that assesses the discrepancy between the task state estimate and the task target, is crucial. This update can involve update in the internal model that estimates the task state from the efference copy of the motor commands. The manuscript tackles a number of important issues in speech control motor. The paper is well-structured, and in most of the sections precisely and clearly written. The addressed topics are interesting primarily for the research community working on speech production and speech perception, but also more generally for all the scientists interested in adaptation mechanisms in human motor control. It also represents an important amount of work. The willingness to share the results of this research work by making programs written in Python available to the community also deserves to be highlighted. However, in our opinion, at this stage, additional information and clarification are needed for a better understanding of the proposed modeling work and, then, to better assess the relevance of the main conclusions. Hence, at this stage, major modifications are required. They do not necessarily require a significant amount of work, but they are in our opinion required for the publication to be considered.*

We appreciate the detailed overview and your feedback.

*The first point to be clarified is the behavior of Design A. The authors wrote that in all three designs “the feedback pathways for online compensation were disabled and direct pathways from articulatory and task state prediction systems were used instead” (lines 133-134). Thus adaptation is modelled from trial to trial by saving the updated predictive/transformation models of trial n and by using them on trial (n+1) (lines 143- 146). However, this strategy does not seem to be systematic, since “auditory feedback or its related information (i.e., the final state estimate updated by state correction signals in Design A and C) were stored in an “error memory, [only] if the auditory prediction errors were larger than a given threshold.” (lines 1043-1045). The authors also added: “We also tested the model simulations with online auditory and somatosensory feedback, but the results were comparable with the simulations presented in the study (i.e., no sensory feedback for guiding movements online).”*

For all three designs, the online feedback pathways (which can affect **ongoing** movement) were disabled. Rather, the sensory feedback or its related information (i.e., state correction signals) were stored and used to update relevant modules **after** each movement, if the size of the prediction errors exceeded a given threshold. In other words, auditory feedback was not used for online compensation in any case. We further clarified this point in the manuscript and revised lines 135-139.

As the reviewer pointed out, we also tested the simulations with online auditory and somatosensory feedback enabled to confirm that our adaptation simulations were not due to online compensation. We also revised the sentence (Lines 1063-1066) to clarify this point:

“In order to rule out the possibility that our findings are limited to the situations without online compensation, however, we also tested all simulations with online compensation and confirmed that these results were comparable with the simulations presented in the study.”

*In this context, the results obtained with design A, as presented in the left panels of Figure 3, require further explanations. Indeed the bottom left panel shows very significant changes in the predicted articulatory state during trials 22, 24, 26, 28, and 30, whereas the difference between the auditory feedback and the auditory prediction remains quasi unchanged across these trials and in any case exceeds the auditory prediction error threshold considered in this study (5Hz, 30Hz, or 50Hz, according to Figure7). According to the above description, if the changes in the predicted articulatory state are associated with a significant update of the internal model that generates the estimation of the articulatory state, the integration of this update from a trial to the next should induce significant changes in the predicted articulatory state (similar to those given in Figure 3) for the unchanged motor commands and this should induce changes in the estimated task state (via the orange box “Task state estimator” in Figure 1) and changes in the output of the “Articulatory state feedback control law”. In turn these changes should induce changes in motor commands and then changes in the auditory output of the model. How to explain then that no adaptation, i.e. no change in motor commands and then no change in auditory output is observed?* *This question is all the more crucial, since the author add that even if the online compensation mechanism is active no adaptation is observed. Since the authors have shown in their former paper (ref 38 in the manuscript) that their model is able to account for online compensation to unpredicted formant perturbation, the problem does not seem to lie in the correction loops. Based on the bottom left panel of Figure 3, it can be considered that the internal model update is taken into account from a trial to the next, since the tongue elevation is different across trials; from trial 10 where this update starts. In addition to the fact that this update from trial to trial does not seem to have any effect on the task state estimation, it is observed that the slope of change in the articulatory state estimation increases from trial to trial, which let the authors speak about “instability”. Hence, all the results presented in the left panels of Figure 3 for Design A, require better analysis and explanations. A* *possible explanation for this somewhat strange behavior could lie in the characteristics of the internal model that estimates the articulatory state and in its update. The calculation of the predicted articulatory state is given in Equation 8 (lines 942-943). It seems to correspond to the integration of a first order ordinary differential equation with constant time steps, which is functionally accounted for via LWPR learning. During a trial with altered auditory feedback, the gradual shift of the articulatory state induced by the Kalman filter is probably associated with a limited local learning of the (first order) regression in a part of the space that is considered to be a so far unexplored region. Could it be that such a local learning is too limited to properly account for the required changes in articulatory state estimation in presence of the applied auditory feedback perturbation? This nevertheless would not explain why the actual tongue state would tend to (very slowly) drift towards the maladaptive direction (Figure 3, left column, top panel, blue line). There are additional questions related to the behavior of Design A as described in Figure 3. Why do the changes in predicted articulatory state not induce changes in auditory prediction, or rather, minute changes in the maladaptive direction (Figure 3, left column, top panel)? Similarly, changes in articulatory-state should result in a change of the predicted somatosensory feedback and then in a somatosensory prediction error. This somatosensory prediction error should induce a limitation of the articulatory state changes induced by the auditory prediction error. However, according to Figure 3, it does not seem to be the case. How to explain these results. Are both sensory prediction errors taken into account with different weights?*

We appreciate the reviewer’s thorough feedback.

(1) why does Design A not result in any noticeable changes in formant output even though auditory errors far exceed the threshold?

We conducted further analyses to examine why Design A shows little to no adaptation. One possibility is that, as the reviewer suggested, because the articulatory state prediction LWPR functions as an integrator for the efference copy (articulatory acceleration), changes in the learned model may be limited to very local contexts. This would be reflected in the model by a large number of receptive fields, each covering only a small area of the input space. However, our analyses showed a the LWPR models contain a very small number of receptive fields, suggesting that the learning is rather broad rather than overly-local.

We conducted a series of additional simulations to follow up on the reviewer’s suggestion that somatosensory signals may have limited the amount of adaptation in Design A. Interestingly, we found that by eliminating or reducing the somatosensory feedback in the state correction (i.e., the articulatory state estimate is corrected based on only the auditory feedback), it is indeed possible to induce a behavior that, at first glance, somewhat resembles learning. Nevertheless, the model behavior is unlike adaptation reported by previous studies in that this design’s adaptive changes are found almost exclusively in the later vowel portions, with minimal changes seen at vowel onset (see Figure A). The ever-accelerating behavior (i.e., velocity increases without reaching a stable position as in Design C) seems to be a sign not of adaptive learning, but rather of unstable control. This instability agrees with simulation results from our initial paper on FACTS, where we found that minimizing or removing somatosensory feedback results in an unstable control in FACTS (Parrell et al., 2019).

To isolate adaptive components of learning rather than potential changes due to model instability, we extracted vowel formants from the early portion of vowel on each simulated trial. As shown in Figure A (right panel), the adaptive effect in these simulations is minimal. Adaptive changes mostly occurring in later portions of the movement is not consistent with the widely agreed view that adaptation reflects updates in feedforward control.

Figure A Design A simulations with no somatosensory feedback in the state correction. Left: Formant trajectories across time steps for trials 20, 22, 24, 26, 28, and 30 (shown as darker shade to lighter shade). Perturbation was set to begin on trial 21. Compared to the baseline trial (Trial 20), other trials show accelerating behaviors. Right: If the formant frequencies were extracted from the early vowel portion (Time step 11-13), the changes in the first formant frequency is minimal across the trials.

(2) why auditory prediction changes in the maladaptive direction

In Design A, a slight auditory prediction changes towards “maladaptive” direction was indeed observed, as the reviewer pointed out. This phenomenon seems to be caused by the non-optimal articulatory state estimate updates in the Jaw Height parameter. In the full model, the jaw height is being overestimated (see Figure B, solid lines), even though F1 in the auditory feedback was increased, which would correspond to lower jaw height. The maladaptive state estimate causes maladaptive prediction changes. In the auditory-only model discussed in our response above, the jaw height is correctly underestimated (see Figure B, dashed line) and the direction of the auditory prediction changes is correct (see pink line in Figure A, right). A potential reason for why jaw height is misestimated in the full Design A model may that the articulatory state prediction was implemented as multiple LWPR models each functioning as an integrator for a single articulatory dimension (i.e., Maeda input parameter). It is possible that the model for Jaw height may be (incorrectly) influenced more by somatosensory feedback than auditory feedback.

Figure B The articulatory state estimate for the Jaw height parameter in two different versions of Design A. The thicker black solid line represents the baseline trial. The subsequent 9 perturbatoin trials are shown in different shades (early trials in darker shades). Dahsed lines: a version of Design A in which the state correction was based on auditory feedback only. Solid line: the full Design A in which the state correction was based on both auditory and somatosensory feedback.

To summarize these results, Design A shows minimal adaptation because the somatosensory feedback, a necessary component for stable control, limits the articulatory state prediction changes, leading to minimal adaptation. However, even when somatosensory feedback is taken out of the model, the architecture in Design A yields changes in articulatory and vowel formants that increase throughout vowel production, unlike the adaptation observed in human participants, which is observable at vowel onset. This is potentially due to non-optimal functionality of , the LWPR-based integration models . Additionally, due to the somatosensory feedback’s contribution, the state correction for some articulatory parameters can occur in the “wrong” direction, leading to maladaptive auditory predictions. Together, these findings further support our original proposition that Design A is not a suitable design for adaptation in the FACTS framework. We addressed the reviewer’s questions in the manuscript as supplemental information (see S1).

*Finally, why did the authors interpret the articulatory state changes depicted in Figure 3 as “instability” (See caption of Figure 3) whereas they tend to reflect proper Kalman integration of a biased feedback in a system that does not learn?*

We removed the wording.

*The second point concerns Design B. In this design, the update concerns only the auditory prediction: the Kalman filter induces changes in the relationship between the articulatory state and the predicted auditory feedback so as to match the predicted feedback to the altered auditory feedback. This update induces no change in the predicted articulatory state, which in turn should, on the next trial, induce no change in the estimated task state and in the output of the articulatory state feedback control law. Therefore, there is no reason for motor commands to change and no adaptation is observed. However, as a result the auditory feedback is not at all what is expected for the intended vowel /ε/. It seems strange to suggest that solely this auditory prediction would update, as if auditory prediction error were entirely ascribed to a problem in the auditory feedback pathway! In fact the behavior of Design B could be deduced from a simple analysis of how the Kalman filter works in this design. We therefore wonder whether the presentation of this design is really relevant from the point of view of speech motor control, unless the authors want to show the limitations of implementing a Kalman filter in this context. In particular, these results do not seem to discard the general relevance in adaptation of the update of an internal model that predicts auditory feedback.*

We agree with the reviewer that auditory prediction alone cannot drive adaptation by design. Although this finding may seem obvious to experts in computational modelling, some researchers have argued that prediction-based forward model updates alone may result in learning (see discussions in Hadjiosif et al., 2021). In fact, from previous conversations we’ve had at motor control conferences, we think that it may be important to demonstrate that it seems unlikely that adaptation can be driven solely by updates in sensory prediction. Thus, the point of demonstrating Design B was not to show limitations of implementing a Kalman filter or discard the general relevance of updating internal models.

*Indeed, it is quite likely that a similar implementation of the Kalman filter in a model where the target would be in auditory space, and not in the constriction space of the vocal tract, would induce an adaptation. Indeed Tourville and colleagues mentioned successful adaptation modeling based on such an update in the DIVA model (Tourville, J. A., Reilly, K. J., & Guenther, F. H. (2008). Neural mechanisms underlying auditory feedback control of speech. Neuroimage, 39(3), 1429-1443) and Patri and colleagues have also modeled in this way adaptation to altered auditory feedback in the GEPPETO model (Patri, J. F., Diard, J., & Perrier, P. (2019). Modeling sensory preference in speech motor planning: a Bayesian modeling framework. Frontiers in Psychology, 10, 2339), and more generally in any model in which auditory targets contribute to the specification of the task (Patri, J. F., Perrier, P., Schwartz, J. L., & Diard, J. (2018). What drives the perceptual change resulting from speech motor adaptation? Evaluation of hypotheses in a Bayesian modeling framework. PLoS computational biology, 14(1), e1005942). More generally the whole paragraph that is devoted to the evaluation of the role of auditory prediction (lines 278-293 and Figure 5) could benefit from being more nuanced and limiting the interpretations to the strict framework of the model and to the scope of evaluation used in this manuscript. For example the authors observed that adaptation was faster when in Design C they “artificially fixed the auditory prediction to remain at the baseline value” (line 284), in other words when the auditory prediction does not respond to the changes in the predicted task state induced by the Kalman filter. This is quite a straightforward consequence of the way the Kalman filter works: ignoring the consequence of adaptation in the auditory prediction, will tend to maintain large auditory prediction errors and then strong feedback based correction. However, does it not generate a risk of instability in adaptation, with a possibly infinitely long adaptation due to the risk that the auditory-prediction error could remain large? More generally the proposed analysis of the role of auditory prediction in adaptation is only valid for the task specification used in the model. As mentioned above with the results obtained with the DIVA and the GEPPETO models, it can be expected that any other model in which the task would be specified in the auditory domain would lead to different conclusions. It would be nice to consider more explicitly this alternative perspective.*

As the reviewer mentioned, the DIVA and the GEPPETO models update internal models (e.g., changes in the auditory-motor target map), which induces adaptation. This is fundamentally different from FACTS, so we agree that the role of auditory prediction in adaptation found in FACTS is only valid within our framework. We added alternative perspectives in the Discussion section:

It should be noted that auditory prediction changes being a source of incomplete adaptation is not consistent with findings from other prominent models of speech production. In DIVA, the internal model updates do not alter the intended auditory target (which is viewed as a form of auditory prediction in their framework, see discussions in Parrell \& Houde). In GEPPETO, even though the auditory-motor internal forward model is updated during learning, the prediction in the auditory domain does not contribute to incomplete adaptation (Patri et al., 2018; Patri et al., 2019).

Hadjiosif, A. M., Krakauer, J. W., & Haith, A. M. (2021). Did we get sensorimotor adaptation wrong? Implicit adaptation as direct policy updating rather than forward-model-based learning. Journal of Neuroscience, 41(12), 2747-2761.

Patri JF, Perrier P, Schwartz JL, Diard J. (2018). What drives the perceptual change resulting from speech motor adaptation? Evaluation of hypotheses in a Bayesian modeling framework. PLoS computational biology, 14(1):e1005942. doi:10.1371/journal.pcbi.1005942.

Patri JF, Diard J, Perrier P. (2019) Modeling Sensory Preference in Speech Motor Planning: A Bayesian Modeling Framework. Frontiers in Psychology, 10:2339. doi:10.3389/fpsyg.2019.02339.

*The third point concerns Design C. The results obtained with this design are great and this is clearly to the credit of the authors. However their proposed interpretation requires further justification. The authors wrote: “[…] although the mathematical compositions of the task SFC law does not change as a result of the task transformation updates, the output of the task SFC law change due to the updates in the transformation. Following this line of reasoning, it could be argued that the core change that yielded adaptation was the control policy (i.e., task SFC law) being “updated” per se.” (lines 478-482). As a matter of fact, this is not fully correct: the control policy does not change; but the predicted task state changes. According to the above mentioned studies in DIVA and GEPPETO adaptation to altered auditory feedback can be properly modeled if the task space is in the auditory space, and if feedback-based correction changes the predicted auditory feedback, which does not affect in any way the control policy. Hence, we rather believe that the results obtained with Design C, as compared to Design A (Design B being not really relevant in our opinion, see above), are due to the fact that the update applies to the internal model that predicts the task state, whether it be constrictions in the vocal tract as in FACTS or regions in formants space as in DIVA or GEPPETO.*

*Furthermore, Design A could give similar results as Design C, since task state depends on articulatory state, if the relationship between the motor commands and the articulatory state were not as simplified as they are in FACTS, thus precluding significant plasticity at this level. If the authors disagree it would be good if they could provide more convincing support for their interpretation.*

We agree with the reviewer’s interpretation that “the results obtained with Design C, as compared to Design A (Design B being not really relevant in our opinion, see above), are due to the fact that the update applies to the internal model that predicts the task state, whether it be constrictions in the vocal tract as in FACTS or regions in formants space as in DIVA or GEPPETO.” In fact, we present a perspective consistent with the reviewer’s interpretation (line 461-463) in the discussion section, which is that adaptation resulted from updates to the internal models that predict the task state. We revised the section to emphasize this perspective by adding that despite the design differences across different models (e.g., DIVA, GEPPETO), the internal model updates that drive adaptation affect task-level control in all of these models:

“Task-level changes leading to adaptation has previously been reported by other computational models. Most notably, models of adaptation in DIVA and GEPPETO both demonstrated that adaptation can be produced through updates in the relationships between articulatory and task spaces, which are defined as the auditory space in these models (forward models of articulatory-acoustic relations in GEPPETO, desired articulatory trajectories in DIVA). Even though the task space in FACTS is defined in terms of constrictions, our results replicated this basic finding. In sum, the importance of task-level updates is highlighted in the FACTS simulations, congruent with both speech and non-speech motor control literature.”

However, there is some room for other interpretations given that the Articulatory-to-Task transformations, strictly speaking, is not a part of either task- or –articulatory-space control modules. For example, the transformation LWPR is not a part of state estimators, nor is it a part of the task state feedback control law. Nevertheless, it is clear that updates to Ariculatory-to-Task transformation change task-level control along with other changes (e.g., auditory prediction). We agree with the reviewer that viewing such control changes as task control law updates may not be precisely correct. Hence, we re-phrased our perspective which views the core mechanism as updates to components involved in task-level control and forward predictive models. We have revised this alternative perspective in the discussion section.

Regarding Design A, we agree that it may be possible to induce adaptation if the relationship between the motor commands and the articulatory state were implemented differently. Though that is theoretically possible, it may also introduce some instability, as mentioned above, due to the articulatory state estimate diverging from the actual state. We have added this information in the supplemental information (S1 Appendix).

*III. Minor points*

*Page 3, lines 48-49.*

*The authors wrote: ”However, errors can drive adaptation even in the absence of corrective movements in both speech [7] and non-speech [32, 33] domains.”*

*This sentence could be clarified. In [7] and [32] the authors considered online corrections. Movement corrections did occur from trial to trial. In [33] movements were planned, and the results do not discard the possibility that the efference copy of motor commands was corrected to modify sensory predictions.*

We realize there was some confusion due to the phrasing in this sentence and have revised it accordingly to clarify our main focus, which was that adaptation can and does occur even in the absence of overt feedback-based corrections to ongoing movements.: “However, sensory errors can update future behaviors without online feedback-based correction in both speech and non-speech adaptation.”

*Page 5, lines 167-170.*

*The authors wrote: “The articulatory state data across time steps showed that the articulatory state estimates started to diverge from the true state as the forward articulatory prediction model was updated (Fig 3, bottom left), likely driven by the up-shift F1 perturbation causing the estimator to under-estimate the true tongue height.”*

*First it would be helpful in all the bottom panels of this figure to know the link between the parameter value in the Y-axis and the tongue height. Since the bottom right figure illustrates adaptation to the F1 up-shift, it can be guessed that a decrease of the parameter is associated with an elevation of the tongue, which is the way to counteract F1 increase.*

Thank you for the suggestion, we added extra notation and texts in Fig 3 to better show the link between the parameter value in the Y-axis and the tongue height.

*In this context, the general trend of the results represented in the bottom-left figure 3 makes sense: the articulatory state estimated via the Kalman filter changes so as to lower the tongue in order for predicted auditory feedback to get closer to the altered actual auditory feedback. Hence, why do the authors consider that the estimator “under-estimate the true tongue height”?*

We agree that the direction of estimate makes sense. A larger Maeda tongue parameter means lower tongue height (i.e., larger constriction), and thus, higher F1. In the bottom-left plot of Figure 3 shows that the tongue parameter was larger than its true state, meaning that the tongue height was under-estimated (i.e., estimated with a lower position/larger constriction) and F1 was over-estimated. Thus, this agrees with the reviewer’s point that predicted feedback was getting closer to the altered feedback.

*Page 15, lines 537-540.*

*The authors wrote: “In other words, adaptation extent in FACTS was limited by changes in auditory prediction and its error threshold. To our knowledge, this Idea has never been examined before and remains to be investigated in future studies.” Is the concept of auditory error threshold different from the concept auditory acuity in speech perception?*

Yes, we revised the first sentence to “Our finding that adaptation extent was limited by changes in auditory prediction (in addition to the error threshold, see the discussion below) in FACTS may be applicable to other models of speech motor control such as Bayesian GEPPETO, in which the auditory-motor internal forward model is updated during learning. Hence, this idea remains to be investigated in future empirical studies.”

We then added additional discussion regarding the error threshold in “Perceptual acuity and auditory noise” subsection of Discussion:

“A computational model, DIVA, has also demonstrated that the auditory acuity defined as the size of auditory targets is negatively correlated with adaptation (Villacorta et al., 2007).”

*Page 15, lines 561-567*

*The authors wrote: “Second, there may be biomechanical reasons why changing the tongue position to counter the F1 shift also changes F2. However, this latter point is not supported by our previous study with the Maeda model that showed a nearly full coverage of the vowel space in the Maeda model output ([78] and also see S1 Appendix). This means that, at least for the target vowel used in the current study (/E/), the adaptive behavior in FACTS should not be constrained by the kinematics of the Maeda model (i.e., articulatory-level changes can certainly be made to alter F1, but not F2).”*

*We understand the line of thinking of the authors. However, as a matter of fact, studies of sensitivity functions in the vocal tract show that any change in vocal tract area function induces simultaneous changes in F1, F2 and F3…*

We revised the paragraph to incorporate the reviewer’s comment:

“Second, there may be biomechanical reasons why changing the tongue position to counter the F1 shift also changes F2. **In fact, studies of sensitivity functions in the vocal tract show that any change in vocal tract area function induces simultaneous changes in F1, F2 and F3 (e.g., Story, 2006).** Nonetheless, it is theoretically possible for the Maeda model to produce a nearly full coverage of the vowel space, suggesting that, at least for the target vowel used in the current study (/E/), the adaptive behavior in FACTS should not be constrained by the kinematics of the Maeda model (i.e., articulatory-level changes can certainly be made to alter F1, but not F2).”

Story, B. H. (2006). Technique for “tuning” vocal tract area functions based on acoustic sensitivity functions. The Journal of the Acoustical Society of America, 119(2), 715-718.

*Page 16, section “Inter-trial variability »*

*It would be interesting if the authors could tell more about the conceptual difference between the notion of target noise and the notion of target regions as used in DIVA or GEPPETO.*

We have added further discussion on how other models like DIVA and GEPPETO introduce target noise. “It should be noted that other computational models of speech implement target noise as well. One example is GEPPETO, the optimal motor control model that controls a biomechanical model of the tongue, was recently re-developed within a Bayesian framework. In the model, motor control variables were randomly selected under a probability distribution, introducing inter-trial variability (Patri et al., 2015), a concept analogous to the target noise in the FACTS framework. In DIVA, although target noise is not explicitly specified, auditory targets are defined as time-varying regions, which allow for articulatory variability within these regions (Tourville et al., 2011).”

Patri, J. F., Diard, J., & Perrier, P. (2015). Optimal speech motor control and token-to-token variability: a Bayesian modeling approach. Biological cybernetics, 109(6), 611–626. <https://doi.org/10.1007/s00422-015-0664-4>

Tourville, J. A., & Guenther, F. H. (2011). The DIVA model: A neural theory of speech acquisition and production. Language and cognitive processes, 26(7), 952–981. https://doi.org/10.1080/01690960903498424

*Reviewer #2: This study refines the authors’ previous computational model of speech (FACTS) in order to account for a range of phenomena in sensorimotor adaptation of speech. A number of versions of the model are proposed, with ones incorporating a task update module being able to yield adaptation patterns similar to the ones seen in literature. This is an interesting model potentially very useful to the field. Limitations to the model are fairly outlined and discussed. Resolving some of them would make for a more comprehensive and helpful model (e.g. modeling how and why adaptation plateaus); however, the model formulations proposed here already provide interesting insights. I found parts of the modeling/results a bit hard to read – minimizing jargon and adding clarifications could help the reader (especially people outside the field, or less familiar with this kind of computational modeling) follow this work more easily. I have a few questions and requests for clarification below:*

We appreciate the reviewer’s feedback. We have limited the use of abbreviations (i.e., state feedback control for SFC) and added clarifications for some instances (i.e., adaptive UKF for AUKF).

*(1) The introduction ends with stating that the paper will “Examine the idea that adaptation is driven by sensory prediction errors” – presenting it as the main question. However, that’s not what’s yet answered here - the model does show how sensory prediction errors could drive speech adaptation, but it does not consider alternatives to that. I think this work would benefit from a discussion of how other kinds of errors (such as task errors) could play a role. For example, in the reaching adaptation literature, there is evidence that task errors are involved in adaptation – for example, by interacting/contributing to implicit visuomotor adaptation along with sensory prediction errors (Leow et al., EJN 2018); more recent work suggested that task errors could drive adaptation on their own (Lew et al., J Neurosci 2000; Rajan et al, “motor performance prediction error” MLMC meeting 2000), without the need for sensory prediction errors. Would be useful to discuss how the model could distinguish which kind of errors drives speech adaptation, either in its current formulation or in the future.*

We agree with the reviewer’s point that there are certainly other types of errors that can drive implicit learning in reaching. As the reviewer pointed out, Leow et al. (2018) demonstrated that removing task errors by “moving the target mid-movement to align with cursor feedback of hand position” reduced adaptation (or unlearning during after-effects). In speech, although adaptation seems entirely implicit (e.g., Lametti et al., 2020; Kim & Max, 2021), adaption can be reduced if the adaptive behavior results in changes in lexical status of the target sound (Bourguignon et al., 2014). Therefore, it is plausible that task errors play a role in speech auditory-motor adaptation, in addition to prediction errors.

Hence, our model is consistent with these findings in that both prediction and task error signals are incorporated in the task-level control law that compares the current task state estimate and the target state. That is, even though we only tested sensory prediction errors-driven adaptation in this study, the design that showed adaptation was clearly the one in which error signals made changes in task-level. It should also be noted that Design C is directly in line with a recent study cited by the reviewer which suggested that Motor Performance Prediction Error (MPPE), the discrepancy between the estimated action and movement goals (Ranjan et al., 2020), drive adaptation.

Per reviewer’s suggestion, we added and expanded this point in both the introduction and discussion sections. In the introduction section of the revised manuscript, we have inserted an additional phrase to fully clarify that more work needs to be done to examine whether task errors can drive speech adaptation (“Although it remains to be elucidated whether other types of errors such as task errors can also drive speech adaptation, we investigated potential mechanisms through which auditory prediction errors…”). We also revised a paragraph in the Discussion section to reflect that task-level control changes are in line with the recent upper limb reaching studies (Ranjan et al., 2020):

“This finding is particularly interesting in light of multiple recent upper limb motor learning studies suggesting that implicit adaptation may be influenced by task errors in addition to prediction errors [10–15]. Likewise, Ranjan & Smith (2020) argued that implicit adaptation could be better explained by error signals that account for changes in both the prediction error-based estimate and the target [70]. Thus, the authors demonstrated that the driving error signals can be described as motor performance prediction errors (MPPE), a discrepancy between the estimated action and movement goal, rather than sensory prediction errors. Directly in line with these perspectives, the task state feedback control law in FACTS computes the discrepancy between the task state estimate and the task target. As a result, in theory, FACTS can adapt from both prediction error and task error. For example, if the task estimate is different from the task target due to the updates in task estimate, the control law issues motor commands to reduce the discrepancy (e.g., Fig 4). Similarly, if the task target is shifted, control law would also issue corrective motor commands for the errors to be reduced (i.e, moving task estimate towards the shifted target). Hence, the FACTS design offers a plausible hierarchical state feedback control framework in which both prediction errors and task errors can contribute to adaptation. This is especially important given that studies continue to examine how task errors or task-space variables may impact speech adaptation (e.g., anchoring sound target, [71], lexical status of the target, [72]).”

Bourguignon, N. J., Baum, S. R., & Shiller, D. M. (2014). Lexical-perceptual integration influences sensorimotor adaptation in speech. Frontiers in Human Neuroscience, 8, 208.

Kim KS, Max L. Speech auditory-motor adaptation to formant-shifted feedback lacks an explicit component: Reduced adaptation in adults who stutter reflects limitations in implicit sensorimotor learning. The European Journal of Neuroscience. 2021;53(9):3093–3108. doi:10.1111/ejn.15175.

Lametti DR, Quek MYM, Prescott CB, Brittain JS, Watkins KE. The perils of learning to move while speaking: One-sided interference between speech and visuomotor adaptation. Psychonomic Bulletin & Review. 2020;27(3):544–552. doi:10.3758/s13423-020-01725-8.

Leow LA, Marinovic W, de Rugy A, Carroll TJ. Task errors contribute to implicit aftereffects in sensorimotor adaptation. The European Journal of Neuroscience. 2018;48(11):3397–3409. doi:10.1111/ejn.14213.

Ranjan T, Smith M. Implicit motor adaptation is driven by motor performance prediction error rather than sensory prediction error. In: Proceedings for MLMC 2020; 2020.Available from: https://sites.google.com/view/mlmc-proceedings/acmcproceedings

*(2) It doesn’t seem surprising that the original formulation – and its two variants, A and B – would not yield adaptation by sensory prediction error: if the forward models (updated through acoustic error as in B, or not as in A) do not include a way for the updated prediction to translate to changed motor output, how could one get any adaptive changes in output?*

*Related to the above: Figure 3, design A: what is the source/mechanism behind the (small) shift in model output here?*

We agree with the reviewer that it not surprising that design B did not yield adaptation. Although this may seem obvious to some researchers, our group noticed that many others continue to think that forward model updates may result in state estimate changes leading to adaptation. As the reviewers pointed out, our simulation clearly shows that there is no way for “the updated prediction to translate to changed motor output” as the reviewer suggested. Thus, we think it is still important to include this design as a part of comprehensive exploration of various adaptation mechanisms for the sake of completeness, even if it is obvious to some readers that the design would not result in adaptation.

Regarding Design A, we thought that articulatory state estimate would be updated to be different from the actual articulatory state, which would result in corrective (adaptive) behavior. As the reviewer points out, we did see such changes, but only in a minimal manner (~1 or 2 Hz changes). The slight shift in the model can be seen in the figure. Not only is this small change too small for adaptation, it is also not consistent within each vowel (each trial). That is, the model’s adaptive behavior increases **throughout** the movement, reaching the maximum towards the end of the vowel. This is unlike empirical data in which participants’ vowel trajectories show stable and consistent production within each trial. Please see also our response to a similar point from Reviewer 1 and we also added supplemental information (S1 Appendix) regarding Design A.

*(3) The point that simulation results are in line with direct policy adaptation (lines 492-495) is interesting – but isn’t that a natural consequence of the model design? The model takes SPE and directly adapts the policy at the task level. By removing updates to the forward model, the SPE never gets reduced, resulting in policy updates that would be, in theory, unlimited (i.e. without any forgetting factors or other way to have a saturation limit, adaptation would keep going beyond the target frequency – akin to an error-clamp in reaching adaptation (Morehead et al., 2017). To distinguish between forward- vs. policy-based adaptation, it would be fair to simulate a true forward-based model as well – where the prediction is updated, and then “inverted” somehow to yield the motor command. I thought that was the articulatory feedback control law would do: I’m a bit puzzled how changing the articulatory feedback control law doesn’t yield adaptation (Figure 5). Isn’t the control law the inverse of the articulatory-to-task transformation, thus assuming that the articulatory-to-task prediction is properly adapted, inverting it would yield adaptive changes at the task level?*

Thank you for the feedback. We agree that adaptation was a consequence of the model design, and this was crucial because we simply did not find other designs that yielded in adaptation. As the reviewer suggested we do not have a true, invertible forward model, and thus our results alone cannot provide direct support for the direct policy adaptation hypothesis, as the reviewer suggested. Nevertheless, by our model design, it is clear that task-level control had to be updated to yield adaptation, a phenomenon generally consistent with the idea of direct policy adaptation. We changed our wording to accurately reflect this – by emphasizing that Design C resulted in adaptation by changing task-level control.

Regarding the figure 5, we agree with the reviewer that the lack of adaptation is puzzling. One reason for the lack of adaptation may be that the articulatory control law is not the “true” inverse of the articulatory-to-task transformation in our framework, as the reviewer pointed out. We used a Moore-Penrose pseudoinverse matrix of the Jacobian in the Articulatory State Feedback Control Law, which is not a unique solution and other formulations might make a difference. In fact, we conducted additional analyses to assess the accuracy of these Jacobians. We found, surprisingly, that the Jacobian matrices had large (and unnecessary) changes in the lip parameters even though such changes are not “optimal” for adaptation. Hence, even when the Artic-to-Task LWPR can successfully update its prediction, its updated Jacobian matrix may not be optimal for the Articulatory State Feedback Control Law. An alternative possibility may be that the task state is more directly related to the acoustic output in that vowel formants are related to constrictions in the vocal tract rather than articulatory positions. Thus, the articulatory state is most similar to somatosensory feedback in this framework (Design C), wherein any articulatory-level changes are further removed from the acoustic output changes, compared to task-level changes. From these findings, although we cannot conclude that articulatory-level changes cannot yield adaptation in other model designs, we show that within the existing FACTS architecture, it is only task-level changes, and not articulatory-level changes, seem to yield adaptation. We have addressed this in the supplemental information (S2 Appendix).

*(4) Was there any reason behind using only the sound /ε/ for simulations? How robust would the model be in predicting adaptive changes when other sounds are being perturbed?*

We chose to simulate the vowel /ε/ because it is one of the most extensively studied vowels in F1 adaptation studies. Given that the adaptive mechanisms in the FACTS model have no linguistic constraints, we think that the result should be robust for other vowels as long as the speech target and potential articulatory changes are not constrained by the Maeda model’s physical constraints (e.g., adaptation to perturbations of vowels near or at the limits of the plant production range may be impossible). A thorough exploration of various vowels in adaptation remains to be investigated both experimentally and computationally. On a related note, as we mentioned in the discussion section, it remains to be examined whether F1 and F2 perturbation in the same direction (e.g., both F1 and F2 increasing) would result in realistic adaptation simulations in FACTS. We revised the discussion section to better address these unknown factors per the reviewer’s comment.

“/ε/ was chosen because the vowel is one of the most extensively studied vowels in F1 adaptation studies. It should be noted that adaptive mechanisms in the FACTS model have no linguistic constraints, but a thorough exploration of various vowels in adaptation remains to be investigated both experimentally and computationally (see Discussion).”

*Other points*

*Line 329: missing citation of commonly reported online compensation response*

Thank you for finding the mistake, we added references:

In agreement with this idea, online compensation response is commonly reported to be much smaller (10% of perturbation, Raharjo et al., 2021) than the adaptive response (30% of perturbation, Kitchen et al., 2022).

Kitchen, N. M., Kim, K. S., Wang, P. Z., Hermosillo, R. J., & Max, L. (2022). Individual sensorimotor adaptation characteristics are independent across orofacial speech movements and limb reaching movements. Journal of neurophysiology, 128(3), 696–710. <https://doi.org/10.1152/jn.00167.2022>

Raharjo, I., Kothare, H., Nagarajan, S. S., & Houde, J. F. (2021). Speech compensation responses and sensorimotor adaptation to formant feedback perturbations. The Journal of the Acoustical Society of America, 149(2), 1147. <https://doi.org/10.1121/10.0003440>

*Lines 599-601: That’s a valid argument, but an explicit report does not necessarily mean that what is being reported was the result of an explicit process.*

Thank you for the feedback. We revised the sentence to “Although explicit report does not necessarily mean that what is being reported was the result of an explicit process, it is possible that such acuity measure may differ from the actual sensorimotor mechanisms involved in adaptation…”

*Lines 609-610: in FACTS design… in FACTS (repeated)*

Thank you, we revised the sentence.

*Lines 707-711: Does online compensation (in absolute terms) decrease, or does it decrease relative to the perturbation size?*

It decreases relative to the perturbation size. To clarify the sentence, we revised it to “Interestingly, even the amount of online compensation also proportionally decreases as the perturbation size increases...”

*Lines 818-820: repeating the previous sentence*

We have removed this sentence, thank you.

*Repository link doesn’t seem to work*

Thank you for catching this, the repository is now open.

*A list of abbreviations (or reducing their number) may be helpful*

We appreciate the reviewer’s feedback. We have limited the use of abbreviations (i.e., state feedback control for SFC) and added clarifications for some instances (i.e., adaptive UKF for AUKF).
