# Supplementary material for: Mechanisms of sensorimotor adaptation in a hierarchical state feedback control model of speech
Source: PLoS Comput Biol. 2023 Jul 28;19(7):e1011244. doi: 10.1371/journal.pcbi.1011244 (PMC10434967; doi:10.1371/journal.pcbi.1011244)
Supplement: Attachment — Submitted filename: ResponseToReviewers_R2.docx [file pcbi.1011244.s006.docx]

**Authors’ responses to the review of PCOMPBIOL-D-22-01400, Mechanisms of sensorimotor adaptation in a hierarchical state feedback control model of speech. Corresponding Author: Kwang S. Kim, Ph.D.**

Note: Below, all of the editor’s and reviewers’ comments are reproduced verbatim in non-indented paragraphs with *blue italicized font*. Our responses are provided with black non-italicized font.

Reviewer's Responses to Questions

**Comments to the Authors:
Please note here if the review is uploaded as an attachment.**

Reviewer #1: I would like to thank the authors for their very interesting responses to the points raised in the review of the preceding manuscript. The changes that they have provided to the text improve the quality of the paper and give place to a more objective and nuanced interpretation of their results. The responses have in particular shed light on the behavior of Design A, in which the absence of adaptation was quite surprising. These explanations should be better integrated in the manuscript, and the discussion should consider them more explicitly. In addition, these insights raise new questions about Design A and Design C that should also be clarified. This is why major modifications are still required, before the manuscript can be accepted for publication.
First of all, reading the comments of the second reviewer, and the answers of the authors to the both reviews, I think that we all agree that Design B is scientifically not relevant. Anyone minimally aware of how SFC works can predict the results of Design B in the absence of a task specification in the auditory domain. This result does not demonstrate that it is wrong to assume that “prediction-based forward model updates alone may result in learning (see discussions in Hadjiosif et al., 2021)” (Citation from the authors’ response). Indeed if the task is defined in the auditory domain, as shown for example in Patri et al., (2018, Plos Comp. Biol.) prediction-based forward model updates does result in learning. Hence, I think that Design B should be removed from the manuscript. It adds unnecessarily to its complexity, and removing it will avoid statements that are too rapid and then inaccurate as in line 185 “These results clearly indicate that updates in auditory prediction did not generate adaptation.”

Thank you for the comment. Our understanding is that Patri et al. (2018) did update forward predictive model to drive adaptation, but critically used the auditory-motor internal model for generating motor commands/planning (essentially, finding a motor command that maximizes the probability of producing the auditory and somatosensory signals associated with a particular phoneme). In this case, because a motor command was generated from inverting the updated forward model, such a model architecture generated learned behavior (adaptive motor command). In contrast, within the FACTS framework, a sensory forward model update *does not directly affect* motor commands (or task state). Thus, regardless of how task is defined in FACTS, we do not believe that updating a forward model module alone would result in adaptation. Given this fundamental architectural difference between FACTS and GEPPETO, we think it is important to explicitly demonstrate that if forward model updates do not directly affect motor commands (but only changes sensory prediction, unlike Patri et al. (2018)), such a model design does not yield adaptation. We agree with the reviewer that some readers who are familiar with the SFC framework may be able to come to the same conclusion without testing the idea, but most readers would benefit from learning about our finding. In fact, it is worth noting that even some of us initially thought that such a design could potentially lead to adaptation prior to testing it.

We further revised the line 185 and added additional information to clarify our point:

“These results indicate that updates in auditory prediction alone did not generate adaptation in FACTS. Although it has been shown that updates to forward models in other frameworks can yield adaptation (e.g., Patri et al., 2018), it is important to note that such models used updated forward models to influence the motor command, leading to adaptation. In Design B, forward model updates did not directly affect motor commands, and our results clearly demonstrate that such a model design does not yield adaptation.”

More interesting is the analysis of the results obtained with Design A.
A first very important result that should absolutely be presented in the text, and not in Supplementary Material (S1 in this case), is the key-role of the somatosensory input in the observed absence of adaptation. This result shows that the key problem in Design A is not the fact that adaptation primarily influences the predicted articulatory state, but that (1) the predicted articulatory state is satisfactory for one of the sensory comparison (the somatosensory one) and (2) the weight of this input is too large to let the auditory prediction error induce significant changes in the articulatory state. Clearly then, adaptation would happen in Design A, if the weight of the auditory modality would be larger than it is in the current implementation. This statement is all the more important since Design C not only integrates a prediction of the Task State, but also distributes the role of the somatosensory and auditory comparison over two different correction mechanisms: the somatosensory comparison, which results in no error, applies to the predicted articulatory state only, whereas the auditory comparison, which results in an error, applies to the predicted task state. It would be interesting to test what would happen in Design C, if the task state would be corrected under the influence of both sensory modalities….
A second important result is the fact that when auditory feedback alone in taken in consideration the adaptation observed in Design A is too slow. This slow adaptation is also observed in the predicted articulatory state in the middle left panel of Fig. 3, which explains why the predicted auditory output (top panel, pink curve) does not change much, since it is measured on the very beginning of the produced vowel. This slow adaptation has a striking similarity with the slow adaptation of Design C (Fig3, top right panel, blue curve), which the authors note is at odds with the experimental data (black curve), and which they corrected by replacing the usual Kalman filter design by the adaptive uscented Kalman filter (AUKF) design (Fig. 4., panel B). What would happen in Design A if AUKF would be used? This is an interesting question that should be tested…
In sum the information that the authors provided in their response about the behavior of Design A is crucial, and cannot be put as an additional information. It has to be in the main text. It clearly suggests that adaptation can occur under certain conditions with Design A. For this adaptation to occur, it requires a certain weighting between the somatosensory and the auditory feedback, and this is an issue that has been already addressed in many papers in the context of the DIVA model, the Bayesian GEPPETO model or of experimental studies such the one of Feng et al. (2011) or Lametti et al. (2012). Now if the speed of the adaptation is not consistent with experimental observations, independently of the design of the Kalman filtering, then this is an important point that speaks in favor of Design C. However, if it is the case, given the fact that, as nicely acknowledged in the revised manuscript in lines 456 to 462, DIVA and GEPPETO demonstrated adaptation in responses to auditory feedback perturbation, when internal models were updated and the task was defined in the auditory domain, it would be probably more correct to change the main conclusion. Indeed the work would then rather show that adaptation is more efficient when updates affect internal models that the task state, rather than any other signal that is not directly related to the task. In this case, the abstract, the discussion and the conclusion should be modified accordingly.

We thank the reviewer for the additional suggestions. We implemented Design A (with no somatosensory feedback contribution) with AUKF, and found that even then the design did not result in additional adaptation (see Fig. 1). Clearly, this is different from Design C which produced clear adaptation along with stable productions. In fact, it should be noted that the tuning of AUKF had to be carefully adjusted for this simulation because of some productions going out of bounds (i.e., unrealistic articulatory state), leading to “breaking” of the FACTS model productions. These results further support our statement from the previous response (“The ever-accelerating behavior (i.e., velocity increases without reaching a stable position as in Design C) seems to be a sign not of adaptive learning, but rather of unstable control....” ).

This figure shows that Design A does not yield adaptation even when equipped with AUKF. We added an additional figure to show this Design A-AUKF simulation in Supporting Information (S1).

To summarize, we have demonstrated that adaptation simulation from Design A “is not consistent with experimental observations, independently of the design of the Kalman filtering” as the reviewer suggested. First, even with AUKF (and no somatosensory feedback), Design A showed minimal changes in F1 across the trials when examining the beginning of the vowel. Second, the within-vowel productions accelerate across the vowel, resulting in unstable productions. We agree with the reviewer that it would be important to list different deviants of Design A that have been explored, so we added an additional paragraph in the main text, summarizing Supporting Information (S1):

“Nevertheless, the true state (i.e., the actual movement) changed minimally across the perturbation trials, leading to minimal changes in the acoustics.

We also explored several other variations of Design A, but none of them simulated adaptation. Relying solely on auditory feedback (by removing somatosensory feedback), for example, resulted in a behavior that first looked like adaptation, but each vowel production had unstable behavior in which the articulators never reached a stable final position. Such within-vowel formant changes are not consistent with empirical data. In addition, applying another type of Kalman filter also did not solve this problem. These results demonstrated that Design A, regardless of other factors such as the amount of reliance on auditory feedback, was not able to simulate adaptation.”

The question raised by Reviewer 2 about the absence of adaptation when the articulatory feedback control law changes in Fig. 5 and the explanation provided by the authors in Supplementary Material S2 should also be clearly integrated in the text. Indeed it shows that the absence of adaptation is not associated with the principle of a change of the articulatory feedback control law per se but with its specific implementation in the context of the Maeda’s model and the computation of the Jacobian matrix used by the authors….. In my opinion the sentence in line 317-320 should then be modified to be clear about this point, and exclude any kind of generalization to other models, even to SFC models that would use another articulatory model or compute the Jacobian Matrix differently.

Thank you for the suggestion. We removed the sentence in line 317-320 and implemented the Supporting Information (S2) into the main text. The revised manuscript now states:

“One reason for this finding may be that we used the Moore-Penrose pseudoinverse (which finds the minimum Euclidean norm solution to a system of linear equations with multiple solutions) of the Jacobian to approximate the true inverse Jacobian in the Articulatory State Feedback Control Law. Further, we found that the Jacobian matrices had surprisingly large changes in the lip parameters that appeared irrelevant for adaptation. Hence, even when the articulatory-to-task LWPR can successfully update its prediction, its updated Jacobian matrix may not capture the appropriate dimensions for the Articulatory State Feedback Control Law. An alternative possibility may be that the task state is more directly related to the acoustic output in that vowel formants are related to constrictions in the vocal tract rather than articulatory positions. In this framework (Design C), the articulatory state is most directly associated with somatosensory feedback.”

Reviewer #2: Thank you for addressing my comments. There definitely interesting issues to be explored further: examples include the issue of saturation of adaptation, or whether using a different/more robust way to invert the articulatory-to-task transformation (discussion in Supplementary materials 2) would change the outcome in Figure 5 Right. However, I find my comments adequately addressed, and that the paper does a good job presenting this interesting model and fairly discussing its limitations.

We agree with the reviewer that it would be interesting to explore further whether adaptation can be simulated with a different or more robust way to invert the articulatory-to-task transformation. We added a sentence in the Discussion section to better reflect this point.

“In the current study, we used LWPR models for all learnable modules such as forward models and articulatory-to-task transformation. However, it remains possible that implementing alternative models such as Deep Neural Network (DNN) could result in different findings. For example, a more robust way to invert the updated articulatory-to-task transformation may yield more adaptation. Future studies should test these possibilities.”
